# *In silico *investigation of lactoferrin protein characterizations for the prediction of anti-microbial properties

**Published:** 2014-06

**Authors:** Seyyed Mohsen Sohrabi, Ali Niazi, Mahmood Chahardoli, Ali Hortamani, Payam Setoodeh

**Affiliations:** 1 Institute of Biotechnology, Shiraz University, Shiraz, Iran; 2 School of Petroleum and Chemical Engineering, Shiraz University, Shiraz, Iran

**Keywords:** Bioinformatics tools, Mammalian species, Anti-microbial activity, Lactoferrin, Lactoferricin, *In silico *study

## Abstract

Lactoferrin (Lf) is an iron-binding multi-functional glycoprotein which has numerous physiological functions such as iron transportation, anti-microbial activity and immune response. In this study, different *in silico *approaches were exploited to investigate Lf protein properties in a number of mammalian species. Results showed that the iron-binding site, DNA and RNA-binding sites, signal peptides and transferrin motifs in the Lf structure were highly conserved. Examined sequences showed three conserved motifs which were repeated twice in the Lf structure, demonstrating ancient duplication events in its gene. Also, results suggest that the functional domains in mammalian Lf proteins are Zinc finger, Tubulin/FtsZ, GTPase, α/β hydrolase and Zinc knuckle. The potential site for nucleic acid binding and the major DNA and RNA- binding sites in this protein were found in the lactoferricin (Lfc) fragment. Due to its high positive charge, Lf is able to bind a large number of compounds. Our analysis also revealed that the interactions between Lf and ITLN1, LYZ, CSN2, and CD14 proteins played an important role in the protective activities of Lf. Analysis for the prediction of secondary structures indicated that high amounts of α-helix, β-strand and β-sheet were present in Lf. The high degree of conservation among mammalian Lf proteins indicates that there is a close relationship between these proteins, reflecting their important role.

## INTRODUCTION

Lactoferrin (Lf) is a non-hemic iron-binding glycoprotein of the transferrin family. Lf is expressed in most biological fluids and tissues and is a key component of the innate immune system. It is a secretory molecule that links innate and adaptive immune systems in mammals [1]. Because of its wide dispensation in diverse tissues, Lf is a multi-functional protein that participates in numerous physiological functions, including: iron transportation, immune response, anti-oxidant, anti-carcinogenic and anti-inflammatory properties, and defense against microbial invasion [2].

Lf gene sequences are highly conserved between distinct species, with an almost same arrangement and an mRNA of about 1900-2600 bp [3]. Lf is a 75-80 kDa glycoprotein containing 700-720 amino acids with high homology between species. It includes a polypeptide chain folded into two regular lobes (N and C lobes) which are highly homologous with each other (between 33–41% homology). The lobes are linked by a joint region comprising parts of α-helix in the Lf, which confers flexibility to the molecule [4]. Lf is a positively charged protein, with a physiological pH of about 7, and an isoelectric point of 8.0–8.5. It contains a number of cysteine residues that allow for the formation of intra-molecular disulfide bonds. Asparagine residues in the N- and C- lobes prepare several N-glycosylation sites [5, 6]. Lf is able to bind iron, copper, zinc and manganese ions; it has also been observed to be bound to lipopolysaccharides (LPS), lipoteichoic acid, heparan sulfate (HS), DNA and RNA [2, 7-12].

Several roles have been identified and described for Lf. The structural properties of Lf provide the functionality beyond the iron homeostasis function common to all transferrin families. Moreover, Lf has strong anti-microbial activities against gram positive and gram negative bacteria as well as fungi, yeasts, viruses [7], and protozoa [13]. It also has anti-inflammatory, anti-tumor [14] and multiple enzymatic activities [15]. The anti-microbial activity of Lf is generally caused by two mechanisms. The first is iron absorption in sites of infection, which deprives the microbes of this nutrient, contributing to an anti-microbial effect. The other mechanism is the direct contact of Lf with pathogens. The positive amino acids in the Lf structure can interact with negatively charged molecules on some bacterial, viral, fungal and parasite surfaces, causing cell lysis.

Lf shows anti-viral activity against a broad range of RNA and DNA viruses which infect humans and animals. The anti-viral activity of Lf has not yet been characterized; however, several modes of action have been suggested. One of the most broadly accepted hypotheses is that Lf binds to viral receptors, especially, heparan sulfate. The binding of Lf to heparan sulfate inhibits the early interaction between the virus and the host cell, consequently inhibiting the infection [8, 16].

Lf has anti-fungal activity through a direct interaction with the pathogen. Absorption and iron deficiency is another important anti-microbial mechanism [17]. Lf positive net charge allows it to interact with negatively charged surfaces of many cells of the immune system [4, 18]. It has been suggested that this interaction can activate signaling pathways which result in cellular responses such as activation, differentiation and proliferation.induce apoptosis, stop tumor cell growth and block the transition of tumor cells from the G1 phase to the S phase in the cell cycle. Lf is also able to bind negatively charged phosphatidyl serine on the outer surface of the cell membrane of some tumor cells and disrupt them [4, 18, 19]

Temperate proteolysis leads to the release of the two N and C-terminal lobes. The treatment of bovine Lf with pepsin produces a short peptide with anti-bacterial activity against gram-positive and gram-negative bacteria, in addition to fungi [20]. In 1992, Bellamy et al recognized a fragment of amino acids at the N-terminus that kept its biological function when released from the complete molecule and showed more anti- microbial activity than Lf. This was named Lfc, which comprised of two cysteine residues linked by a disulfide bridge containing many hydrophobic and positively charge damino acids.

The physiological ability of Lf in the immune system together with recent pharmaceutical and nutritional needs led to the classification of Lf as a nutraceutical protein. Two basic approaches are currently used to obtain Lf. First, natural Lf can be isolated and purified from the milk and colostrum of mammals. Second, recombinant Lf can be produced from animal, plant, bacterial and fungal expression systems [5, 21].

In the present work, different features of Lf and Lfc in a number of mammalian species were investigated, using bioinformatics tools to find explanations for their anti- microbial activities.

## MATERIALS AND METHODS

**Multiple Sequence Alignments: **In order to investigate the multiple sequence alignments of Lf sequences, CLCbio 5.8 and Vector.NTI 10.3 were used with the following parameters: gap extension penalty=0.05, gap opening penalty=10.0, and BLOSUM protein weight matrices***.***

**Motif and functional domain identification: **Motifs in Lf were identified, using MEME program [22], run from the web server (http://meme.sdsc.edu/meme/cgi- bin/meme.cgi) with the following parameters for each motif: maximum width 50 amino acids (aa), minimum motif width 6aa, and maximum motif number 15aa. CLC protein workbench tool (www.clcbio.com/protein) based on the Markov model [23, 24] was used to predict secondary structures and functional domains. MAST (http://meme. nbcr.net/meme/doc/mast.html) was also utilized to achieve consensus motifs.

**Phylogenetic tree constructions: **The Molecular Evolutionary Genetics Analysis (MEGA) software (Version 5.0) was used to obtain the molecular evolutionary and phylogenetic analyses of 10 Lf protein sequences in mammals. Molecular distances of the aligned sequences were considered according to the *p*-distance parameter, and the phylogenetic tree was constructed using the Neighbor-Joining method with pairwise [25].

**Protein sequence analysis: **Protein analysis was performed by CAMP database (http://www.bicnirrh.res.in/antimicrobial/pr.php) and Expasy ProtParam tool (http://web.expasy.org/protparam/). Also, the CLC protein workbench tool was utilized to compute protein features such as molecular weight, length, Amino acid frequency, isoelectric point, aliphatic index, hydropathy, electrical charge.

I**nteraction network: **The search for proteins capable of interacting with Lf among all the identified proteins was performed by protein–protein interaction network, using STRING 9 (http://string-db.org) database. Since an almost well-defined protein**–**protein interaction network in *Homo sapiens *was available*, *the Lf protein with the GenBank accession no. AAA59511.1 was used. The interactions included direct (physical) and indirect (functional) associations.

**Secondary structure prediction: **Proteus2 (http://www.proteus2.ca/proteus2/ index.jsp) Servers as well as the CLC protein workbench tool based on the Markov model [23] were used for the prediction of secondary structures.

**Nucleic acids Binding sites and Anti-microbial peptide prediction: **Potential sites for DNA and RNA binding were identified by BindN server (http://bioinfo.ggc.org/bindn/) [26]. The search for an anti-microbial peptide in the Lf structure was performed by CAMP and AntiBP (http://www.imtech.res.in/raghava/ antibp/) servers.

## RESULTS AND DISCUSSION

**Figure 1:**
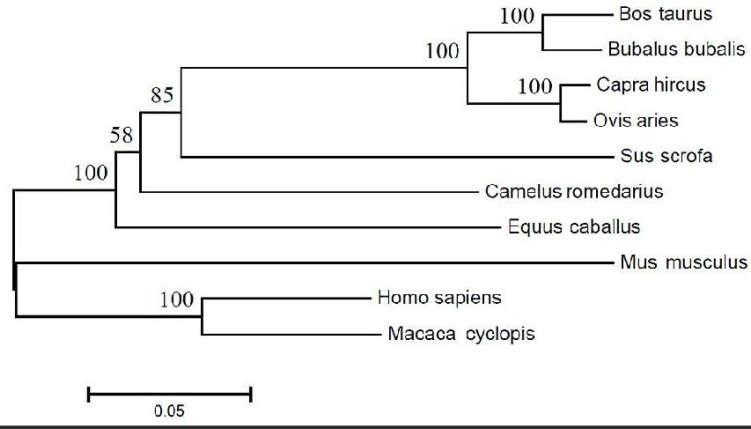
Phylogenetic tree of Lf proteins from different species plotted by MEGA5 software

**Multiple Sequence Alignments and Phylogenies of Lf proteins: **Multiple alignment and identity analysis (Table 1 and Fig. 1) with MEGA5 and Vector NTI software were carried out with Lf protein sequences (experimentally verified sequences) retrieved from the NCBI database.

**Figure 2:**
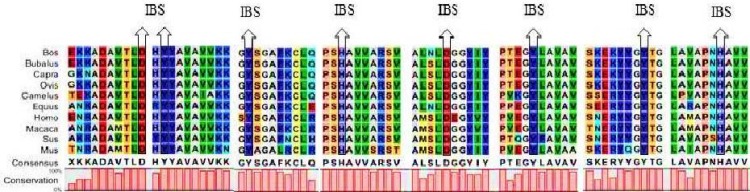
Conserved amino acids in the iron binding site (IBS).

**Figure 3:**
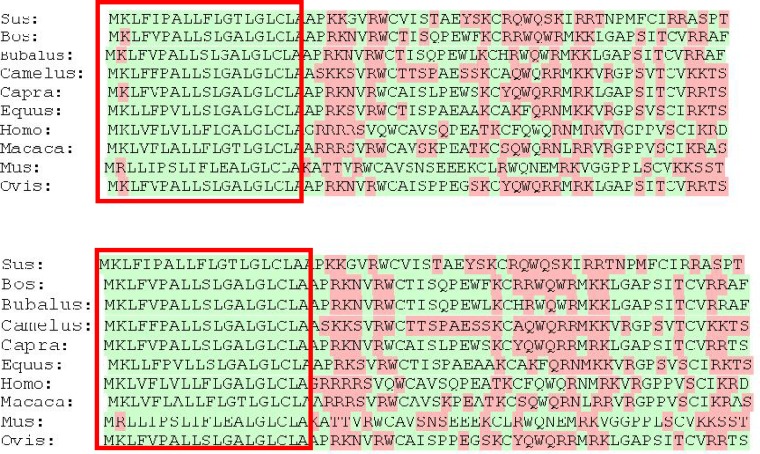
Nucleic acids binding sites and signal peptides in Lf structure. Upper: DNA binding site. Lower: RNA binding sites. Rectangles show the signal peptides

**Figure 4:**
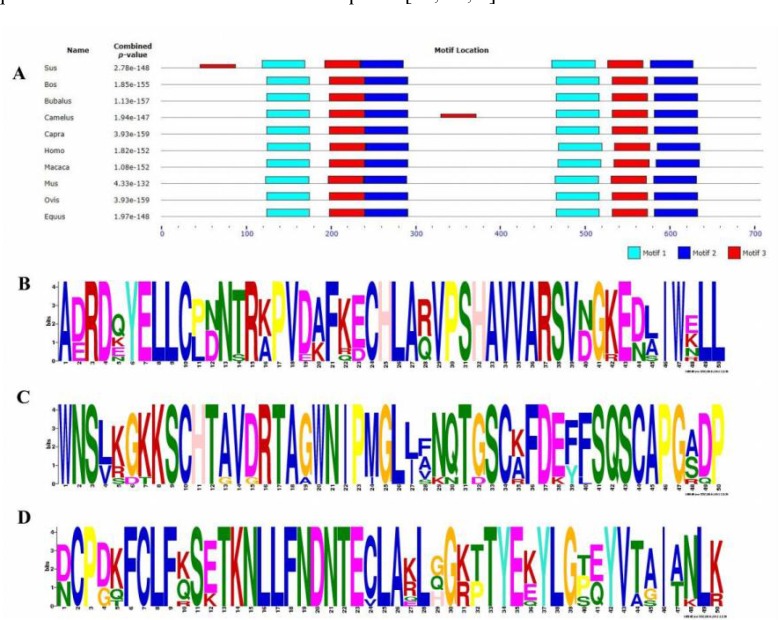
Multiple alignment of Lf motif amino acid sequences in mammalian species using MEME database. A. conserved motif of Lf in mammalians. B (motif 1), C (motif 2) and D (motif 3). Consensus of conserved motifs

These analyses showed that the iron binding site (Fig. 2), DNA and RNA binding sites, signal peptides (Fig. 3) and transferrin motifs (Fig. 4A) were highly conserved between species. Multiple sequence alignment also showed 64-100% identity between species. The sequence relationships revealed that the Lf proteins belonged to the highly conserved family. Also, the analysis of the Lf protein sequence showed eight completely conserved amino acids (Asp60, Tyr92, Tyr192, His253, Asp395, Tyr433, Tyr526 and His595) for iron binding in the Lf structure (Fig. 2).This represents the role of Lf in iron metabolism and anti-bacterial properties stemmed from iron binding and iron deficiency. The phylogenetic tree structure depicts that these proteins are divided into two distinct groups. The first group contains representatives from Bovidae, Camelidae, Suidae and Equidae families and the second one comprises of Hominidae, Cercopithecidae and Muridae families. Members of these two groups are not very diverse and there is a high sequence identity among them. There is no combination of members between these two distinct groups, demonstrating that they separated early in evolution.

**Figure 5:**
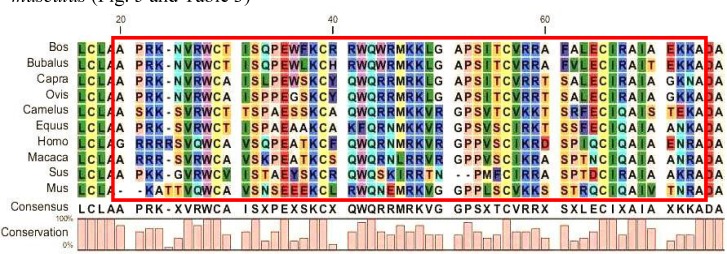
Multiple sequence alignment of Lf in Lfc fragment.

**Motifs and functional domains in Lf proteins: **MEME database was used to identify the conserved motifs in Lf. We found three transferrin conserved motifs in the examined sequences (Fig. 5A). Motif 1 or Transferrin 1 with 50 amino acids’ consensus was from 467 to 516 (Fig. 5C). Motif 2 or Transferrin 2 with 50 amino acids’ consensus was between 241 and 290 (Fig. 5B). Motif 3 or Transferrin 3 with 50 amino acids’ consensus was from 643 to 692 (Fig. 5D). All three motifs were repeated twice in the Lf structure, demonstrating ancient duplication events in the Lf gene [27]. Motif 3 was repeated three times in *Sus scrofa *and *Camelus dromedaries*. Hence, we may conclude that it contributes to the higher activity in iron metabolism. Lf belongs to the transferrin family. It can be concluded, therefore, that in addition to the role of Lf in iron metabolism and its general anti-microbial mechanism with iron modulation, the presence of transferrin motifs is also required [15, 23, 7].

Identification of functional domains in mammalian Lf proteins, carried out with the CLC software, showed that these proteins contain Zinc finger, Tubulin/FtsZ, GTPase, α/β hydrolase and Zinc knuckle functional domains. Buffalos and goats have three domains; cattle, camels, horses and sheep have just one domain, but for humans and mice, no domain was identified (Table 2). The identification of Tubulin domains could notify anti-viral activities of Lf. The successful replication of viruses depends on their interaction with the microtubule network. The trafficking of Lf-filled endosomes and interaction with tubulin may challenge with the microtubule and impress intracellular trafficking or replication of these viruses [28]. Zinc finger proteins are among the most abundant proteins in eukaryotic genomes. They are described by the coordination of one or more Zn2+ ions in order to stabilize the fold. Their roles are phenomenally diverse, including DNA binding, RNA packaging, transcriptional activation, membrane association and lipid binding. Our predictions regarding the ability of Lf to bind to Zn2+ ions and DNA were confirmed; we also found that Lf can interact with bacterial membranes and lipids. Consequently, the associated domains with this activity should be presented in its structure.

**Nucleic acids Binding sites in Lf proteins: **Analysis of Lf protein sequence with BindN and CAMP databases revealed many potential sites for nucleic acid binding. In addition, the major DNA and RNA-binding sites in this protein were found in the Lfc fragment. The presence of a high positive charge at these sites was also confirmed (Fig. 3). The positive or negative charge of a typical protein can change as a function of pH. It should be noted that the net charges of Lf and Lfc in this study were calculated in a physiological pH of about 7. Due to the high positive charge of Lf, it has the ability to bind large amounts of compounds such as lipopolysacharides, heparin and glycosaminoglycans. Moreover, it is capable of binding DNA and RNA viruses [29, 16, 8]. The potent concentration of positive charge constitutes the proposed binding site for DNA [30]. Lf has also been known as a transcription factor, capable of entering a cell and starting the transcription of specific DNA sequences [31]. Lf and Lfc cause the inhibition of replication, transcription and translation of the viral components by binding to viral DNA and RNA.

**Anti-microbial peptide of Lf proteins: **Proteolysis of Lf leads to the release of lactofericin (Lfc) short peptide. This peptide shows anti-bacterial, anti-viral and anti- tumor activities due to its high positive charge and the ability to interact with negatively charged substrates such as glycosaminoglycan, heparan, lipopolysaccharide, phosphatidyl serine and nucleic acids [14, 19]. The search for anti-microbial peptides in the sequence of Lf proteins using the AntiBp server and CAMP database showed that there was one potential anti-microbial peptide in all mammalian Lf (Table 3). Multiple sequence alignment of these peptides showed that there is a relatively high conservation among them (Fig. 5). The high density of basic amino acids as well as the presence of high positive charged and conserved cysteine and tryptophan residues (contributing to the formation of cyclic forms and the stabilization on different surfaces) demonstrate that the probable sequence of this peptide is located in the same fragments as other species. Comparison of the net charge, isoelectric pH (PI) and tryptophan residues of different species shows that these peptides have the highest activity in *Bos taurus*, *Bubalus bubalis *and *Macaca cyclopis *while they have the lowest activity in *Mus musculus *(Fig. 5 and Table 3)

**Figure 6:**
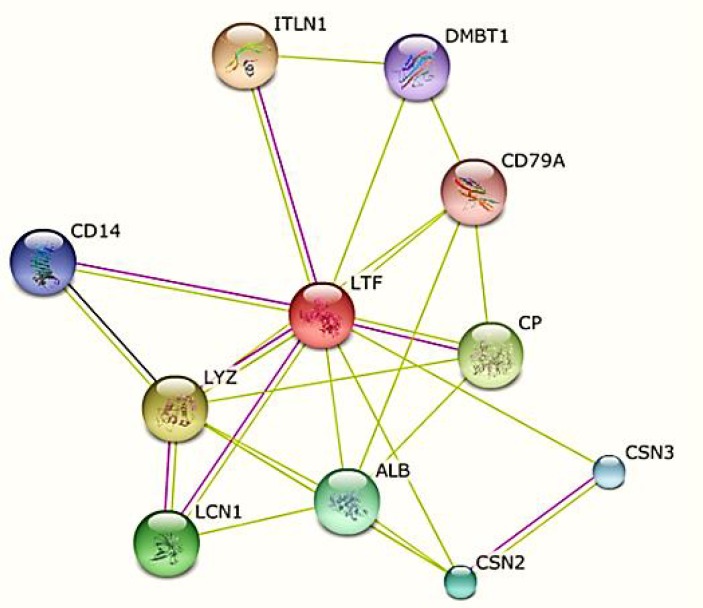
Protein–protein interaction network of all proteins which interact with Lf

**Interaction network of Lf: **A protein–protein interaction network analysis indicated that this protein had many functional partners and interacted with several proteins (Fig. 6 and Table 4). Among the various proteins identified, ITLN1, LYZ, CSN2 and CD14 were selected due to their protective roles and their interaction with Lf in protection activities. Intelectin 1 (ITLN1) is an intestinal Lf receptor. Lysozyme (LYZ) is a part of the innate immune system and has anti-bacterial effects against gram- positive and gram-negative bacteria. Lysozyme is abundant in a number of body secretions. Lf and lysozyme showed co-operative anti-bacterial functions against gram- positive and gram-negative bacteria. Lf can bind to lipoteichoicacid and lipopolysacharide (LPS) on the surface of these bacteria, contributing to better accessibility of lysozyme to bacterial membranes. β-casein (CSN2) is present in high concentrations in milk. Lf and β-casein in milk were shown to have inhibitory functions against bacterial and viral cysteine proteases. The CD14 molecule cooperates with other molecules to mediate the innate immune response to bacterial lipopolysaccharide, cytokine secretion, and inflammatory responses. Lf is able to act as an anti-endotoxin through binding to LPS released from lysed bacteria inhibiting the binding of LPS to CD14 receptors on innate immune system cells and septic shocks. The existence of large amounts of LPS leads to the excessive production of immune mediators, resulting in septic shock. Lf exists in body secretions in relatively high levels and needs to interact with other proteins directly or indirectly to remain active. Our predictions regarding these facts were confirmed [10, 32-35].

**Protein sequence analysis: **Lf protein sequences were analyzed using CLC software, CAMP and ExpasyProtParam databases. The molecular weight of the Lf proteins was estimated to be approximately 75-80 kDa. Moreover, a protein length of

703-711 aa and the net charge of +8 to +21 were calculated. An aliphatic index of 56 to

67 and a hydropathy between -0.24 to -0.42 were determined (Table 5). The hydropathy index of a protein is a number representing the hydrophobic or hydrophilic properties of its amino acids. While negative values of the hydropathy index indicate hydrophilic characters of a protein, the positive values stand for its hydrophobic properties. The aliphatic index of a protein is a portion of the relative volume occupied by an aliphatic side chain of the nonpolar amino acids alanine, valine, leucine and isoleucine. Increases in the aliphatic index add to the thermo stability of globular proteins. Being a globular protein and regarding its calculated aliphatic index, Lf, is expected to have high thermo stability. Also, Lf is a glycoprotein commonly found in body secretions. For iron transport and protection activities Lf has to be relatively soluble in water. Thus, it should have a negative hydropathy index.

Bactericidal effects of Lf have been attributed to its direct interaction with bacterial membranes and cell walls [9]. The positively charged Lf inhibits interactions between lipopolysaccharide and the bacterial cell wall cations, causing the release of lipopolysaccharides from the cell wall and an increase in membrane permeability, hence damaging the bacteria. Lf acts against gram-positive bacteria by binding its net positive charge to negatively charged molecules on the bacterial surface. Lipoteichoic acid, for instance, reduces the negative charge on the cell wall and allows for the action of other anti-bacterial compounds such as lysozyme [10].

The analysis of Lf protein sequences in different species showed a relatively high net charge. *Mus musculus *with +21 had the highest while *Bubalus bubalis *and *Equus caballus *with +8 and +9 respectively had the lowest net charges. The high positive charge in Lf is due to the large amounts of Arg and Lys amino acids in its structure. These amino acids play an important role in most Lf activities such as interacting with and disrupting bacterial membranes and binding to lipids and nucleic acids.

**Table 1:** Identity of Lf protein between mammalian species

**Species**	***Bos***	***Bubalus***	***Camelu s***	***Capra***	***Equus***	***Homo***	***Macaca***	***Mus***	***Ovis***	***Sus***
*Bos taurus*	100	96	75	92	72	69	69	64	92	74
*Bubalus bubalis Camelus*		100	76 100	92 76	73 78	70 74	69 73	64 67	92 76	74 75
*dromedarius*										
*Capra hircus*				100	73	71	70	64	98	74
*Equus caballus*					100	75	74	66	74	72
*Homo sapiens*						100	89	70	71	70
*Macaca cyclopis* *Mus musculus*							100	70 100	71 64	72 64
*Ovis aries*									100	74
*Sus scrofa*										100

**Table 2:** Functional domains in Lf structure.

**Organism**	**Start**	**End**	**Domain function**	**Accession**	**Domain (PFAM)**
Bos taurus Bubalus bubalis	176 388	217 415	Zinc finger. C3HC4 type (RING finger) Tubulin/FtsZ family. GTPase	PF00097 PF00091	zf-C3HC4 Tubulin
Bubalus bubalis	189	217	domain Zinc finger. C3HC4 type (RING	PF00097	zf-C3HC4
Camelus	176	217	finger) Zinc finger. C3HC4 type (RING	PF00097	zf-C3HC4
dromedarius Capra hircus	387	415	finger) Tubulin/FtsZ family. GTPase	PF00091	Tubulin
Capra hircus	176	217	domain Zinc finger. C3HC4 type (RING	PF00097	zf-C3HC4
Equus caballus	189	198	finger) Zinc finger. C3HC4 type (RING	PF00097	zf-C3HC4
Equus caballus	189	217	finger) Zinc finger. C3HC4 type (RING	PF00097	zf-C3HC4
Macaca cyclopis	313	359	finger) alpha/beta hydrolase fold	PF00561	Abhydrolase_1
Mus musculus	186	201	Zinc knuckle	PF00098	zf-CCHC
Ovis aries Homo sapiens	176 -	217 -	Zinc finger. C3HC4 type (RING finger) -	PF00097 -	zf-C3HC4 -
Suss crofa	-	-	-	-	-

**Table 3:** Lfc sequence analysis

Organism	Lactoferricin sequence	PI	Net charge
*Bos taurus*	APRKNVRWCTISQPEWFKCRRWQWRMKKLGAPSITCVRRAFALECIRAI AEKKA	8.43	+11
*Bubalus bubalis*	APRKNVRWCTISQPEWLKCHRWQWRMKKLGAPSITCVRRAFVLECIRAI TEKKA	7.95	+10
*Camelus* *dromedarius* *Capra hircus*	ASKKSVRWCTTSPAESSKCAQWQRRMKKVRGPSVTCVKKTSRFECIQAI STEKA	8.34	+10
	APRKNVRWCAISLPEWSKCYQWQRRMRKLGAPSITCVRRTSALECIRAI AGKNA	8.07	+10
*Equus caballus*	APRKSVRWCTISPAEAAKCAKFQRNMKKVRGPSVSCIRKTSSFECIQAIA ANKA	8.03	+10
*Homo sapiens*	GRRRRSVQWCAVSQPEATKCFQWQRNMRKVRGPPVSCIKRDSPIQCIQA IAENRA	8.17	+9
*Macaca cyclopis*	ARRRSVRWCAVSKPEATKCSQWQRNLRRVRGPPVSCIKRASPTNCIQAIA ANRA	8.23	+12
*Ovis aries*	APRKNVRWCAISPPEGSKCYQWQRRMRKLGAPSITCVRRTSALECIRAIA GKKA	8.07	+11
*Susscrofascrofa*	APKKGVRWCVISTAEYSKCRQWQSKIRRTNPMFCIRRASPTDCIRAIAAK RADA	8.31	+10
*Mus musculus*	ATTVRWCAVSNSEEEKCLRWQNEMRKVGGPPLSCVKKSSTRQCIQAIVT NRADA	8.66	+4

**Table 4:** Predicted functional partners for Lf protein

Protein name	Function	Accession number	Length (aa)	Score
ITLN1	Intelectin1(galactofuranose	AF271386_1	313	0.990
	binding)			
LYZ	Lysozyme (renal amyloidosis)	NP_000230	148	0.989
CP	Ceruloplasmin (ferroxidase)	NP_000087	1065	0.979
LCN1	Lipocalin 1 (tear prealbumin)	NP_002288	176	0.979
ALB	Albumin	NP_000468	609	0.965
CSN2	Casein β	NP_001882	226	0.953
CSN3	Casein kappa	NP_005203	182	0.953
CD14	CD14	NP_000582	375	0.937
DMBT1	Deleted in malignant brain	NP_015568	2413	0.928
	tumors 1			
CD79A	CD79a molecule	NP_001774	226	0.924

**Table 5:** Sequence analysis of Lf.

**Organism**	**Length**	**Weight (kDa)**	**Net Charge**	**Aliphatic Index**	**Hydropathy**
*Bos taurus*	708	78.167	17	77.88	-0.3
*Bubalus bubalis*	708	77.729	8	79	-0.25
*Camelus dromedarius*	708	77.211	15	79.94	-0.25
*Capra hircus*	708	77.339	10	77.9	-0.24
*Equus caballus*	708	75.99	9	75.28	-0.31
*Homo sapiens*	711	78.409	11	74.51	-0.35
*Macaca cyclopis*	710	77.87	12	77.25	-0.3
*Ovis aries*	708	77.282	10	76.94	-0.24
*Sus scrofa*	703	77.512	14	76.06	-0.29
*Mus musculus*	707	77.837	21	72.76	-0.42

**Table 6:** Predicted secondary structure of Lf

Organism	Helix (%)	β-Sheet (%)	Random coil (%)
*Bos grunniens*	22	19	59
*Bos indicus*	19	19	62
*Bos taurus*	21	19	61
*Bubalus bubalis*	20	20	60
*Camelus dromedaries*	24	17	59
*Capra hircus*	20	21	59
*Equus caballus*	20	19	61
*Homo sapiens*	22	19	59
*Macaca cyclopis*	21	20	59
*Ovis aries*	20	17	63
*Sus scrofa*	21	20	59
*Mus musculus*	19	19	62

**Figure 7:**
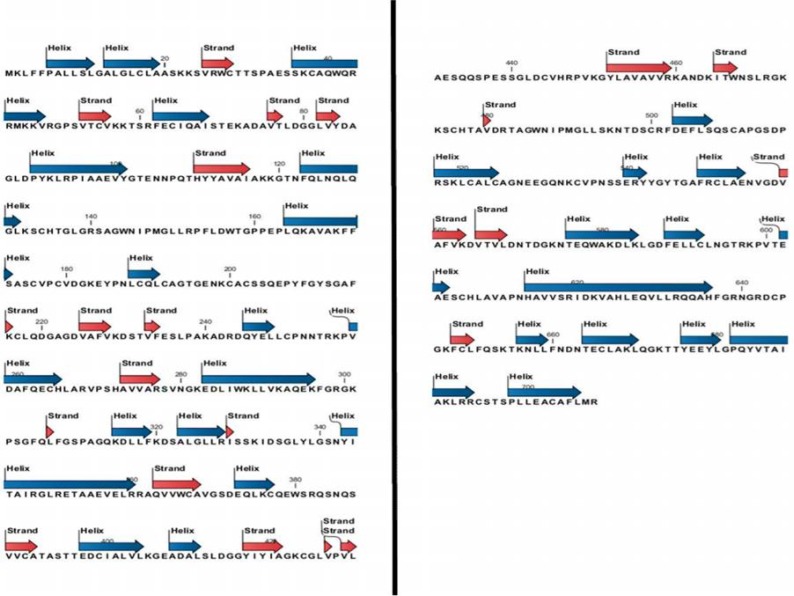
Predicted secondary structure of Lf.

**Secondary structure prediction: **Analyses for the prediction of secondary structures indicated that large amounts of α-helix, β-strand and β-sheet were present in Lf (Fig. 7 and Table 6). Some of the functional roles of α-helix are binding to DNA with helix-turn-helix, leucine zipper and Zinc finger structures as well as interacting with cell membranes. β-sheets are involved in protein-protein interactions. Thus, the existence of these structures in Lf confirms that its function in transcription regulation, anti-viral activities, anti-bacterial activities and interaction with other proteins is inherent. Also, the formation of α-helix in the core of the Lfc peptide leads to increases in the activity of this peptide.

In the present study, Lf protein sequences of some mammalian species were analyzed using bioinformatics methods. Multiple sequence alignment of Lf from various species showed the high conservation existing among these species. The transferrin motifs involved in iron transportation were identified in all Lf proteins. Additional transferrin was observed in *Sus scrofa *and *Camelus dromedarius *species*. *Functional domains were identified in some species such as buffalos, goats, cattle, camels, horses, macacas and sheep, while in humans and mice no domain was observed. The binding ability of nucleic acids was observed in all Lf proteins. Protein–protein interaction network analysis indicated that there were several protein partners for Lf. Some of these partner proteins contributed to the anti-microbial activities of Lf. The molecular weight of the Lf proteins was computed to be approximately 75-80 kDa. Also, a protein length of 703-711 aa, a net charge of +8 to +21, an aliphatic index of 56 to 67 and a hydropathy of -0.24 to -0.42 were calculated. Structural analysis showed that all Lfs comprised of two homologous lobes which had the same fold and were linked by a short α-helix. Each lobe was further divided into two α/β domains. This structural arrangement was shared by all Lfs. Lf is a multifunctional protein involved in a large number of important physiological activities. Due to its unique anti-microbial, immune modulatory and even anti-neoplastic properties, Lf seems to have great potential for medical and pharmacological studies. Up to now, most of the studies conducted on Lf have been limited to special species, such as humans and bovines and its potential advantages for other species has still remained uncovered. It is strongly recommended that more studies be conducted on other types of Lfs.
